# Educational attainment and offspring birth weight: A bidirectional Mendelian randomization study

**DOI:** 10.3389/fgene.2022.922382

**Published:** 2022-09-01

**Authors:** Yu Liu, Chen Jin, Li-Fang Ni, Tian Zheng, Xiao-Chen Liu, Shan-Shan Wang, Hui-Jun Huang, Ming-Min Jin, Bin-Wei Cheng, Hong-Tao Yan, Xin-Jun Yang

**Affiliations:** Department of Epidemiology and Health Statistics, School of Public Health and Management, Wenzhou Medical University, Wenzhou, China

**Keywords:** educational attainment, offspring birth weight, causal association, two-sample bidirectional mendelian randomization, instrumental variable

## Abstract

**Background:** The association between educational attainment (EA) and offspring birth weight (BW) has been reported by several traditional epidemiological studies. However, evidence for this association tends to be mixed and confounded. This study aimed to investigate the causal association between EA of parents and offspring BW.

**Methods:** Here, we carried out a two-sample bidirectional Mendelian randomization (MR) analysis to examine the causal association between EA of males (n = 131,695) and females (n = 162,028) and offspring BW using genetic instruments. Summary statistics of EA associated single nucleotide polymorphisms (SNPs) were extracted from a GWAS incorporating 293,723 individuals of European descent performed by the Social Science Genetic Association Consortium (SSGAC), and the effects of these SNPs on offspring BW were estimated using a GWAS meta-analysis of 86,577 participants of European descent from 25 studies. Univariable MR analyses were conducted using the inverse-variance weighted (IVW) method and four other methods. Further sensitivity analyses were carried out to test the viability of the results. Multivariable MR was used to examine the confounders between the exposure and outcome.

**Results:** The result shows evidence that the offspring BW is positively causally affected by female EA. Each one standard deviation (SD) increase in female EA was associated with 0.24 SD higher of offspring BW (95% confidence interval [CI], 0.10 to 0.37, *p* < 0.001 for the IVW method). Similarly, change in offspring BW was 0.21 SD (95% CI: 0.07 to 0.34, *p* = 2.82 × 10–^3^) per one SD higher in male EA. No causal effect of BW on EA was found by any of the five methods. The causal association between female EA and offspring BW maintained after adjusting for alcoholic drinks per week and BMI. The effect of male EA on offspring BW was attenuated when we adjusted for BMI and alcoholic drinks per week using multivariable MR analysis.

**Conclusion:** Our study indicated that female EA is positively causally associated with offspring BW. The association between male EA and offspring BW may be confounded by alcoholic drinks per week and BMI.

## Background

Birth weight (BW) is a significant sign of fetal development and health, and it is closely related to the long-term disease and health of the fetus. On average, newborns with a BW below the 10th percentile and above the 90th percentile of the gender and gestational age reference population in many developing and developed countries may be at a higher risk of adverse birth outcomes and long-term adverse health conditions, such as stillbirth, neonatal death, and diabetes (Hediger et al., 1999; Spittle et al., 2014; Spittle et al., 2015; Wang et al., 2020). In addition, newborns with abnormal BW are at a higher risk of psychological and behavioral development problems. For example, BW has been reported to have a U-shaped association with problem behavior (Hwang et al., 2019).

Many influencing factors, such as biological, demographic, and socioecological factors throughout pregnancy may affect BW. Educational attainment (EA) is one of the most important components of socioeconomic status. It also plays an important role in health promotion. Researchers from Indiana University showed that spousal EA is positively related to an individual’s overall health, and its impact is even comparable to the impact of education level on one’s own (Halpern-Manners et al., 2022). Therefore, in addition to being valuable to individuals, education is also a shared health resource. Maternal EA plays a role through many intermediaries, and it is one of the most important social determinants of BW (Keenan-Devlin et al., 2021). In addition, it has been reported that higher EA of the father is associated with a lower risk of small for gestational age (SGA) and low birth weight (LBW) (Alio et al., 2010).

The impact of EA of parents on the offspring BW has been widely discussed, but the conclusions are inconsistent. Moreover, studies paid less attention to the influence of paternal factors. Higher maternal and paternal education is associated with a lower risk of LBW (Shapiro et al., 2017; Godah et al., 2021; Guarnizo-Herreno et al., 2021; Shen et al., 2021). However, some studies did not find any association (Vahdaninia et al., 2008; Amegah et al., 2013). From these conventional epidemiological studies, we can find that the associations observed are likely to be confounded by social and environmental factors already existing before birth.

The Mendelian randomization (MR) analysis approach is a new method that examines whether a potential factor is causally associated with outcome. This method uses genetic variation associated with exposure as an instrumental variable (IV) (Lawlor et al., 2008). Compared with traditional epidemiological methods, this approach selects a single nucleotide polymorphism (SNP) that is randomly assigned to individuals along with gametes as the IV to avoid the relationship between exposures and outcomes from being affected by confounding factors such as environmental factors (Davey Smith and Hemani, 2014). Therefore, we carried out two-sample bidirectional MR analyses to detect the causal association between male and female EA and offspring BW.

## Materials and methods

### Study design

**FIGURE 1:**
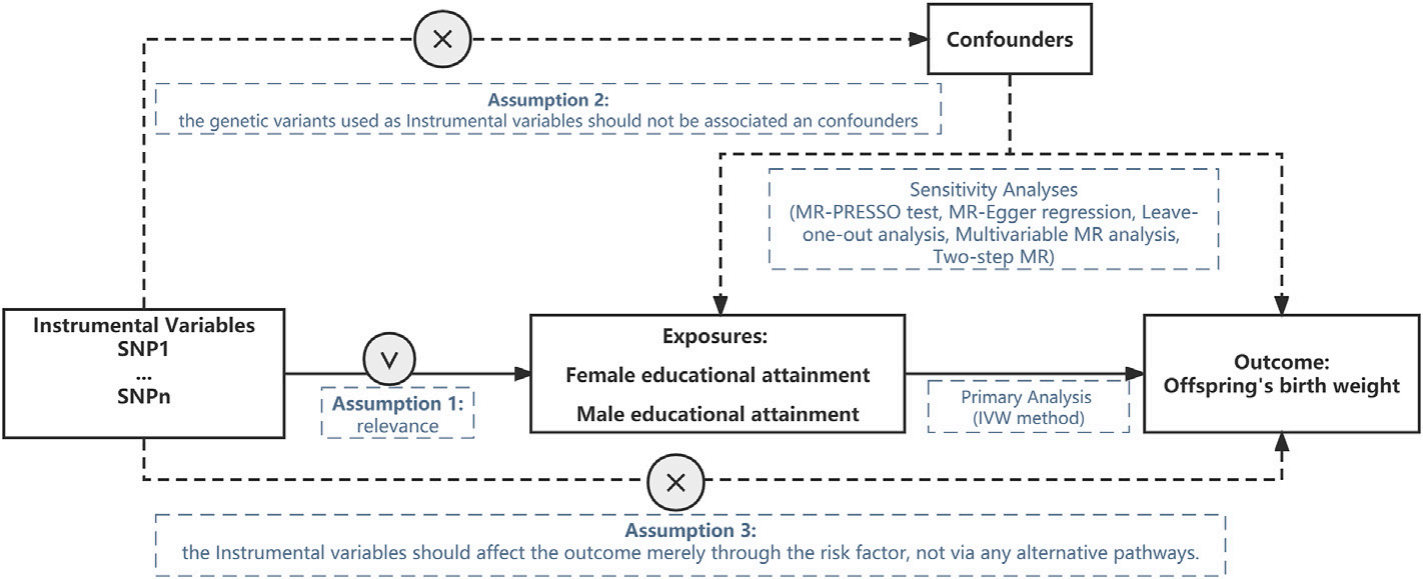
Sketch map for the overall design of the Mendelian randomization study. SNP: single nucleotide polymorphism.

This MR design is based on three basic assumptions (Swerdlow et al., 2016). First, the genetic IVs selected should be strongly associated with educational attainment (Assumption 1), if the correlation between IVs and exposure is weak, weak IVs is likely to occur. To ensure a strong correlation between IVs and exposure factors, the *F*-statistic of each SNP is used to judge the correlation strength and avoid the bias caused by weak instrumental variables. When the *F*-value is greater than 10, it indicates that there is less possibility of weak instrumental variable bias. Second, confounding factors should not affect the selected IVs that impact the association between EA and offspring BW (Assumption 2). In order to satisfy assumption 2, we scanned the Secondary phenotypes of selected SNPs using the GWAS catalog. Secondary phenotype-associated SNPs at the genome-wide significance level were eliminated from the IVs. Five MR approaches were further performed similarly. Third, IVs can only affect the offspring BW through EA (Assumption 3). The second and third assumption are often violated since most genetic variants are actually pleiotropic. Therefore, beyond conventional MR analyses, we conducted a series of robust MR analyses, including Mendelian randomization pleiotropy residual sum and outlier (MR-PRESSO), MR–Egger analysis, Cochran’s Q analysis, The leave-one-out approach, multivariable MR, two-step MR to account for pleiotropic effects. This study used public data, therefore, ethical approval or consent to participate is not needed. Figure 1 shows the overall design of this study. GWAS summary data used in this study was listed in Table 1.

**TABLE 1:** Overview of GWAS data used in MR analysis.

actConsortium	Phenotype	Participants	Author	Year of publication	PubMed ID
SSGAC	Years of schooling	293,723	Okbay et al	2016	27225129
EGG and the United Kingdom Biobank	Offspring birth weight	86,577	Beaumont et al	2018	29309628
GERA	BMI	315,347	Hoffmann et al	2018	30108127
GSCAN	Alcoholic drinks per week	335,394	Liu et al	2019	30643251

### SNPs associated with educational attainment

We obtained the GWAS summary statistics of EA-associated single SNPs from a database. All genome-wide association studies (GWAS) were performed at the cohort level in samples restricted to individuals on 131,695 males and 162,028 females of European descent whose EA was assessed at or above age 30 by the Social Science Genetic Association Consortium (SSGAC) (Okbay et al., 2016). Here, the way we defined education was consistent with this study. The EA is evaluated by the years of schooling finished, according to the International Standard Classification of Education 1997 classification scale (EduYears, mean = 14.3, SD = 3.6). They identified 74 approximately independent lead SNPs associated with the years of schooling completed. To remove linkage disequilibrium effects between multiple IVs, we further performed clumping by setting the genetic distance as 10,000 kb, removing the SNPs with *r* ^*2*^ greater than 0.001 with the most significant SNPs, and retaining the one with *p* ≤ 5 × 10^−8^ in each locus. In this study, we finally obtained 22 and 15 SNPs from the female and male education GWAS summary-level data, respectively, as IVs associated with female and male EA. The *F*-statistic was used to examine the power of IV, and it is a function of the size and precision of their genetic effects.

### Effects of SNPs on offspring birth weight

The effects of these instruments on offspring BW were estimated using a GWAS meta-analysis. This study was performed by Beaumont RN, including 86,577 participants of European descent from 25 studies (Beaumont et al., 2018). For each SNP associated with EA, we extracted summary data from the GWAS database. The study performed by Beaumont RN et al. normalized birth weight, and one SD represented a birth weight of 484 g. Among the 15 SNPs associated with male EA, one SNP had a palindrome structure (rs10740118), so it was excluded from the IVs. The remaining SNPs were available in the BW database, and we did not use proxied SNPs. Finally, the set of 22 SNPs (Supplementary Table S1) representing female EA and 14 SNPs (Supplementary Table S2) representing male EA was kept for offspring BW.

### Mendelian randomization analysis: The causal effect of educational attainment on offspring birth weight

To examine MR estimates for EA on offspring BW, we used several MR approaches. Unless otherwise mentioned, the “*TwoSampleMR*” package was used in R software (version 3.6.3) to carry out the main MR analyses. In this study, we applied standard inverse variance weighted (IVW) estimates for the main analysis, which combined the Wald ratio of each SNP on the outcome and obtained a pooled causal estimate. When all IVs used in Mendelian randomization are valid (and uncorrelated), the IVW method is consistent and most powerful, however, when horizontal pleiotropy is present, it may lead to biased inference (Xue et al., 2021). Four other statistical tests (the weighted median estimator (WM), the simple mode, the weighted mode, and MR–Egger regression methods) are also performed to check the reliability of the estimates. The WM estimator can effectively aggregate the effects of IVs if the weight of valid IVs exceeds 50% (Bowden et al., 2016). MR–Egger regression was conducted to explore whether there was gene pleiotropy (if the intercept term was close to 0) and to examine the causal estimate adjusted for pleiotropy (Bowden et al., 2015). Therefore, with the 5 MR methods based on different assumptions, a comprehensive evaluation of the results is possible.

### Sensitivity analyses

We examined directional pleiotropy based on the intercept by MR–Egger analysis to evaluate the possibility that MR assumptions may be violated. An intercept term close to 0 (*p* < 0.05) indicates that there was no horizontal pleiotropy between the selected genetic instruments. The MR pleiotropy residual sum and outlier (MR-PRESSO) was used to explore horizontal pleiotropy to corrected estimates by eliminating outliers (Burgess et al., 2017). Cochran’s Q analysis was used to test the heterogeneity among the SNPs. The existence of heterogeneity indicates that some genetic instruments are invalid (*p* < 0.05). Fixed effects IVW assumes that each SNP provides the same estimate. Random effects IVW relaxes this assumption, allowing each SNP to have different mean effects. This will return an unbiased estimate if the horizontal pleiotropy is balanced, i.e. the deviation from the mean estimate is independent from all other effects. The estimates from the random and fixed effects IVW models are the same but the variance for the random effects model is inflated to take into account heterogeneity between SNPs. Therefore, the fixed-effect model was used if no heterogeneity was observed (*p* > 0.05); otherwise, a random-effect model was applied (Bowden et al., 2017; Hemani et al., 2018). The leave-one-out approach was used to evaluate if the MR estimate is driven or biased by a single SNP that might have a particularly large horizontal pleiotropic effect. In this method, we can re-estimate the effect by sequentially dropping one SNP at a time. Identifying SNPs that, when dropped, lead to a dramatic change in the estimate can be informative about the sensitivity of the estimate to outliers.

To further rule out confounding of MR results by horizontal pleiotropy, we scanned the Secondary phenotypes of the selected SNPs using the GWAS catalog (http://www.ebi.ac.uk/gwas). Secondary phenotype-associated SNPs (such as associate with BMI, Alcohol intake frequency) at the genome-wide significance level were eliminated from the IVs (Supplementary Table S3; Supplementary Table S4). Five MR approaches were further performed similarly.

### Reverse Mendelian randomization analysis: the causal effect of birth weight on educational attainment

After detecting the causal effect of EA on offspring BW, we performed a reverse MR analysis. In this analysis, the exposure was birth weight, and the outcome was educational attainment. Potential causal estimates of BW for EA were similarly examined by five methods. A series of sensitivity analyses were also performed to test the existence of horizontal pleiotropy and heterogeneity among the SNPs.

### Multivariable mendelian randomization estimates

Multivariable Mendelian randomization (MVMR) is an extension of MR that can explore the causal association of multiple exposures with an outcome at the time. This approach additionally assumes that all selected IVs must be conditionally independent of the outcome, given all exposures and confounders (Sanderson et al., 2019). Studies have found the associations between EA and body mass index (BMI) (Liwin, 2022; Zhang et al., 2022; Zhao et al., 2022), alcoholic drinks per week (Liu et al., 2019; Treur et al., 2021; Zhou et al., 2021). In addition, BMI of parents is well-established influencing factor for offspring birth weight according to both clinical observational and genetic association studies (Vogelezang et al., 2020; Chen S. et al., 2021; Thompson et al., 2021; Aydogan Mathyk et al., 2022; Gootjes et al., 2022). Therefore, we further conducted MVMR including EA, BMI and alcoholic drinks per week as exposures, along with offspring BW as an outcome, to examine whether EA and offspring BW are still statistically associated after adjusting for confounders (Zuber et al., 2020). Summary statistics of BMI and alcoholic drinks per week associated with single SNPs were obtained from the Genetic Epidemiology Research on Adult Health and Aging (GERA) cohort (Hoffmann et al., 2018) and GWAS and the Sequencing Consortium of Alcohol and Nicotine use (GSCAN) (Liu et al., 2019), respectively.

### Two-step Mendelian randomization

If univariable MR results suggest a causal association between EA and offspring BW, based on multivariable MR results, it would make sense to further investigate the role of confounders between EA and offspring BW. Therefore, two-step MR was utilized to assess potential confounding effects. This is a strategy, based on the well-established framework of Mendelian randomization, to interrogate the causal relationships between exposure, confounders and outcome. The two-step approach first uses genetic instrumental variables for the exposure of interest to assess the causal relationship between exposure and confounders. In the second step, then utilizes genetic instrumental variables for confounders to interrogate the causal of these confounders on the outcome (Relton and Davey Smith, 2012). In this study, in the first step, we used genetic IVs for the female and male EA to assess the causal relationship between EA and alcoholic drinks per week, BMI, respectively (IVW method). Reverse MR analysis was performed to further detect the causal effect from genetic IVs of alcoholic drinks per week and BMI to female and male EA. And in the second step the genetic IVs of alcoholic drinks per week and BMI were used to perform MR analysis against offspring BW. Confounding effects were suggested if evidence of causalities appeared in both steps.

## Results

### Causal effect from educational attainment to offspring birth weight

**FIGURE 2:**
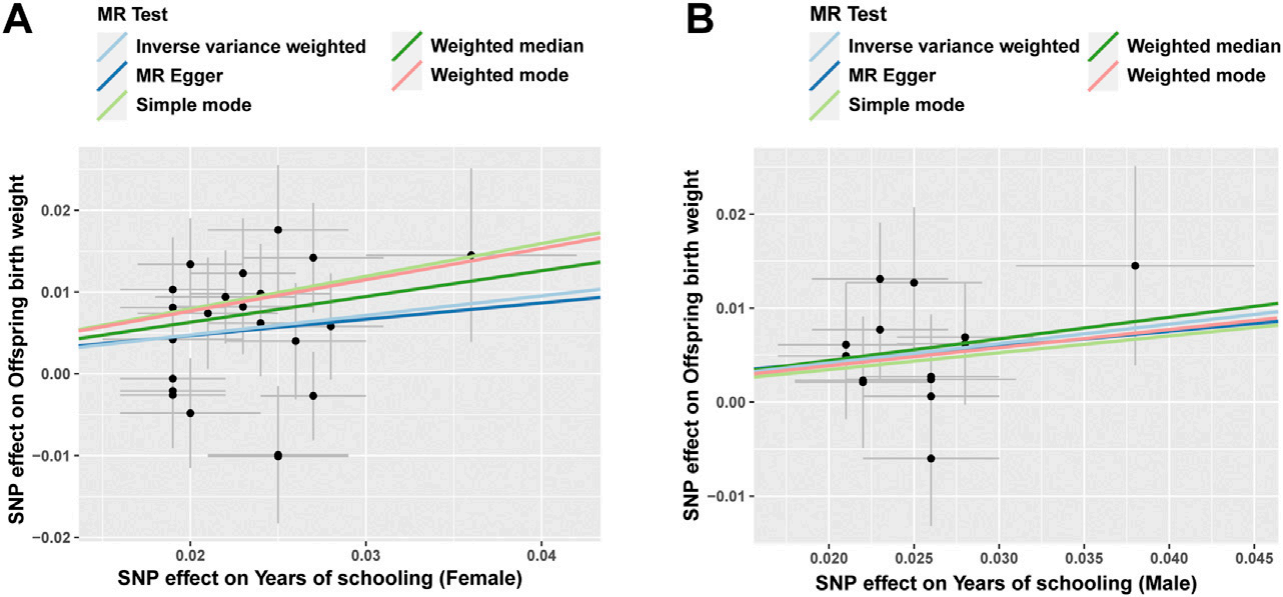
The causal effect of educational attainment on offspring birth weight by five methods. The causal estimates for female educational attainment on offspring birth weight are shown in **(A)**, while the causal association between male educational attainment and offspring birth weight is shown in **(B)**.

**FIGURE 3:**
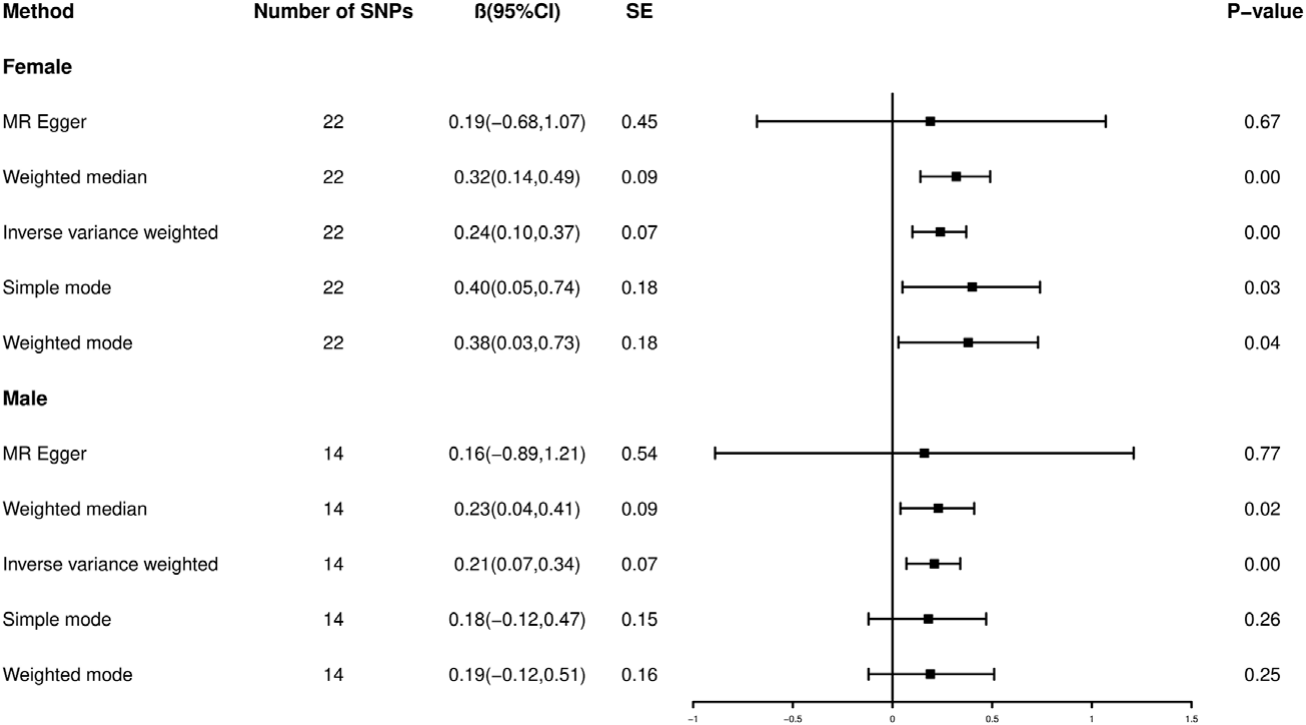
Forest plot showing Mendelian randomization causal effect estimates of educational attainment on offspring birth weight. CI, confidence interval.

Twenty-two SNPs closely related to female EA (*p* < 5 × 10^−8^) and independent of each other (*R* ^2^ < 0.001) were identified (Supplementary Table S1) after analysis. The average *F*-statistic of these SNPs was 35.7537 (range, 25.5765–60.9609), indicating that the results are less likely to deviate due to the influence of weak IVs, which is consistent with assumption 1. We finally used these twenty-two SNPs as IVs to determine MR estimates of female EA for offspring BW. In general, genetically predicted, a higher female EA was associated with a significant increase in offspring BW (Figure 2A). The main MR results (Figure 3) indicated that one SD (3.6 years) longer female EA (across 22 SNPs) was associated with 0.24 SD (1 SD of offspring BW = 484 g) higher offspring BW by the IVW method (95% CI: 0.10 to 0.37, *p* < 0.001). As expected, the MR results were similar to other methods using weighted median (*β* = 0.32, 95% CI: 0.14 to 0.49, *p* < 0.001), weighted mode (*β* = 0.38, 95% CI: 0.03 to 0.73, *p* = 0.04) and simple mode (*β* = 0.40, 95% CI: 0.05 to 0.74, *p* = 0.03), but the estimates provided are not as precise as IVW analysis.

Similarly, fourteen SNPs closely related to male EA (*p* < 5 × 10^−8^) and independent of each other (*R* ^2^ < 0.001) were identified (Supplementary Table 2), and the average *F*-statistic was 33.9043 (range, 24.2159–51.1190). The MR results suggested that genetically male EA was positively causally associated with offspring BW (Figure 2B; Figure 3) (*β* = 0.21, 95% CI: 0.07 to 0.34, *p* < 0.001 for the IVW analysis), and the result of the weighted-median method was consistent (*β* = 0.23, 95% CI: 0.04 to 0.41, *p* = 0.02). Offspring BW increases by 0.21 SD for every SD increase in male EA (across fourteen SNPs) using IVW analysis.

### Sensitivity analysis of educational attainment and offspring birth weight

**FIGURE 4:**
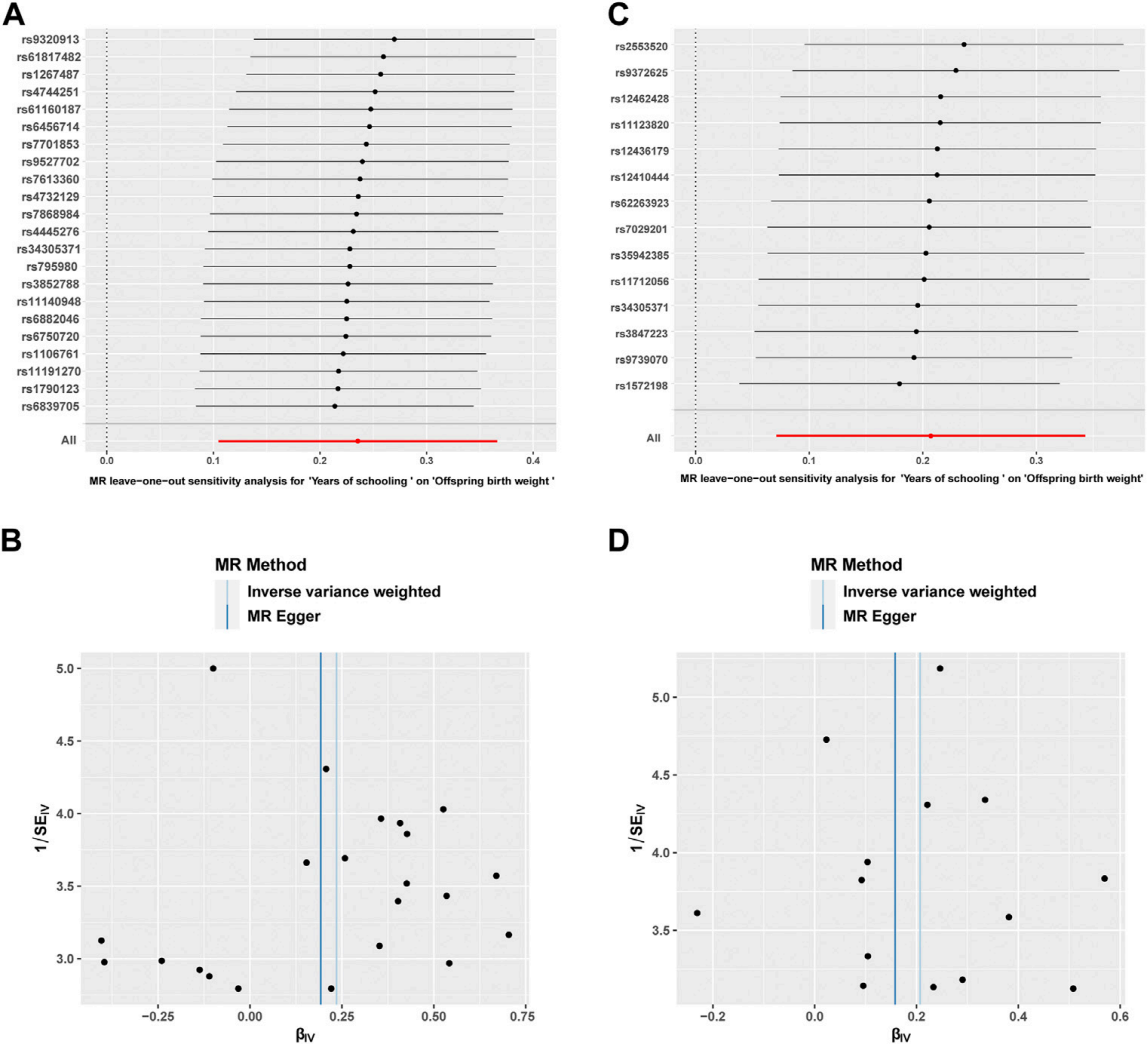
Sensitivity analysis results of leave-one-out plots and funnel plots. The leave-one-out plot **(A)** showing the causal effect of female educational attainment on offspring birth weight after eliminating SNPs one by one. The funnel plot **(B)** displayed the overall symmetry of the causal effects of all instrumental variables. Likewise, sensitivity analysis results of the leave-one-out plot **(C)** and funnel plot **(D)** presented the male educational attainment-offspring birth weight relationship.

The MR–Egger regression and MR-PRESSO approaches were used to explore the existence of horizontal pleiotropy (Table 2). For the sensitivity analyses of female EA and offspring BW, there was little evidence of the presence of horizontal pleiotropy by the MR–Egger test (the intercept term was close to zero, *p* > 0.05). No outlier SNPs or horizontal pleiotropic effects were detected between female EA and offspring BW by the MR-PRESSO test (*p* > 0.05). Additionally, we did not find heterogeneity by Cochran’s Q test (*p* > 0.05). We further conducted a leave-one-out analysis to detect potential SNPs that could confound the MR estimate (Figure 4A). The result shows that the overall MR estimate did not change considerably after eliminating SNPs one by one. Simultaneously, the funnel plot was symmetrical as a whole, which confirmed the insignificant heterogeneity and verified the stability of the result by the IVW analysis (Figure 4B).

**TABLE 2:** The results of Mendelian randomization sensitivity analyses.

Exposures	MR-Egger regression			Heterogeneity test^a^		
	Intercept	Standard Error	*p* value	*Q*-statistic	*df*	*p* value
Female education attainment	9.98 × 10^−4^	0.01	0.92	25.2	21	0.24
Male education attainment	1.24 × 10^−3^	0.01	0.93	7.49	13	0.88

a Heterogeneity test was performed with the fixed-effect inverse-variance weighted approach. MR, mendelian randomization.

Likewise, in the sensitivity analyses of male EA and offspring BW, there was no horizontal pleiotropy between the selected genetic instruments by the MR–Egger test (the intercept term was close to zero, *p* > 0.05) and MR-PRESSO test (*p* > 0.05). Cochran’s Q test showed that there was no significant heterogeneity (*p* > 0.05) (Table 2). The leave-one-out plot indicated that the association of genetically predicted male EA and offspring BW did not change significantly after removing SNPs one by one (Figure 4C). The funnel plot also suggested that the results were robust (Figure 4D).

Then, we scanned about the associations between the IVs and their potential secondary phenotypes (*p* value < 5 × 10^–8^) from the GWAS catalog (date checked January 2022). Four SNPs associated with female EA were associated with other traits (Supplementary Table S3), and the results (Supplementary Figure 1SA; Supplementary Figure S2) did not change substantially after excluding these SNPs (*β* = 0.19, 95% CI 0.03 to 0.34, *p* = 0.02 using the IVW method). By searching the GWAS catalog (data checked January 2022), we found that three out of fourteen SNPs associated with male EA were associated with other traits (Supplementary Table S4), and the results (Supplementary Figure 1SB; Supplementary Figure S2) did not change substantially after excluding these SNPs (*β* = 0.21, 95% CI: 0.05 to 0.37, *p* = 9.1 × 10–^3^ using the IVW method).

### Reverse Mendelian randomization analysis: The causal effect of birth weight on educational attainment

**FIGURE 5:**
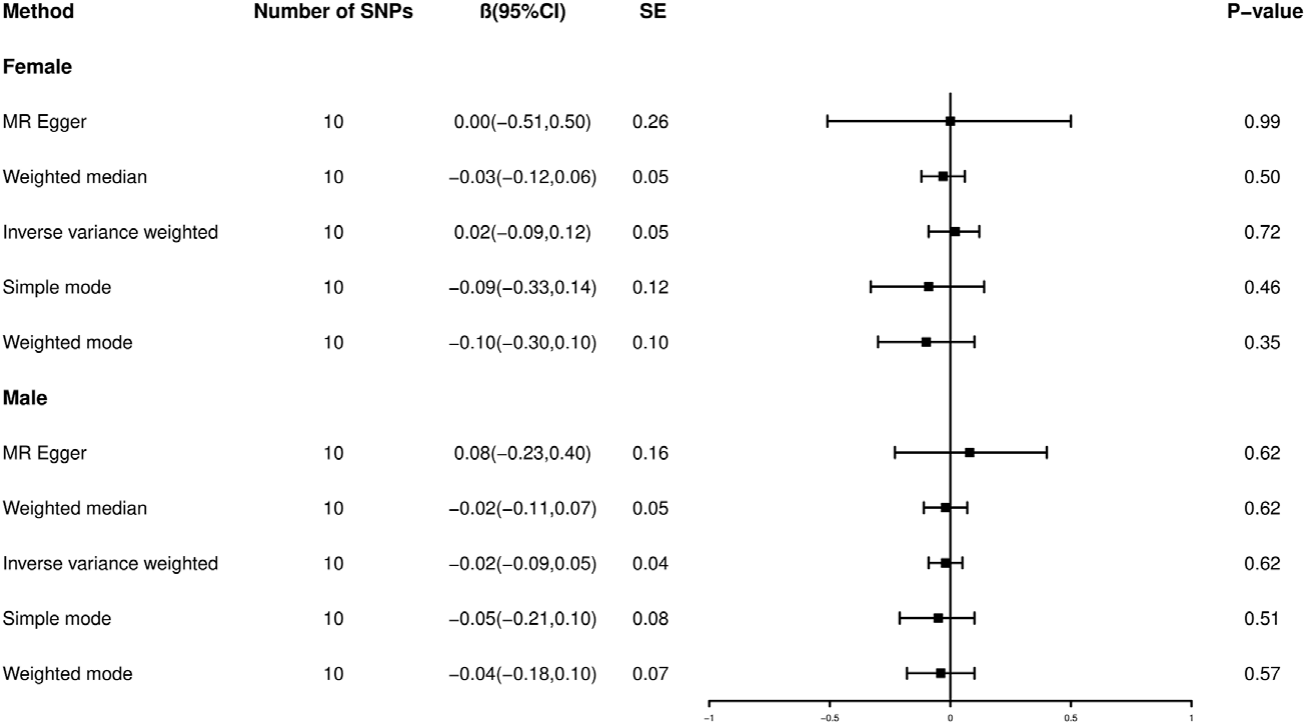
Forest plot showing reverse Mendelian randomization causal effect estimates of birth weight on education attainment.

A reverse MR was conducted to explore the causal effect of BW on EA (Figure 5). Among females, the result of the IVW method showed that BW had no significant causal effect on EA in women (*β* = 0.02, 95% CI: -0.09 to 0.12, *p* = 0.72), and the result was similar to other methods using weighted median (*β* = -0.03, 95% CI: 0.12 to 0.06, *p* = 0.50), weighted mode (*β* = -0.10, 95% CI: 0.30 to 0.10, *p* = 0.35), MR–Egger (*β* = 0.00, 95% CI: 0.51 to 0.50, *p* = 0.99) and simple mode (*β* = -0.09, 95% CI -0.33 to 0.14, *p* = 0.46). Similarly, among males, the causal effect of BW on EA in men was not significant by IVW analysis (*β* = -0.02, 95% CI: -0.09 to 0.05, *p* = 0.62), weighted median *β* of -0.02 (95% CI: 0.11 to 0.07, *p* = 0.62), weighted mode *β* of -0.04 (95% CI: 0.18 to 0.10, *p* = 0.57), MR–Egger *β* of 0.08 (95% CI: 0.23 to 0.40, *p* = 0.62) and simple mode *β* of -0.05 (95% CI: 0.21 to 0.10, *p* = 0.51).

In the sensitivity analyses of BW and EA (Table 3), among females, there was no horizontal pleiotropy between the selected IVs by the MR–Egger test (*p* > 0.05) and MR-PRESSO approach (*p* > 0.05). Cochran’s Q test indicated the existence of heterogeneity in the MR effect estimates (*p* < 0.05). Therefore, we mainly used the random-effect model to estimate the MR effect size, and the multiplicative random effects further demonstrated that BW had no causal effect on female EA (*β* = 0.02, SE = 0.05, *p* = 0.72). Among males, no horizontal pleiotropy effect of BW on male EA was identified by the MR–Egger test (*p* > 0.05) or the MR-PRESSO test (*p* > 0.05). Moreover, Cochran’s statistical test showed no statistically significant heterogeneity effect (*p* > 0.05).

**TABLE 3:** The results of reverse Mendelian randomization sensitivity analyses.

Outcomes	MR-Egger regression			Heterogeneity test^a^		
	Intercept	Standard Error	*p* value	*Q*-Statistic	*df*	*p* value
**Female education attainment**	9.29 × 10^−4^	0.01	0.93	28.17	8	0.00
**Male education attainment**	-4.29 × 10^−3^	0.01	0.53	10.32	8	0.24

a Heterogeneity test was performed with the fixed-effect inverse-variance weighted approach. MR, mendelian randomization.

### Multivariable mendelian randomization estimates

**FIGURE 6:**
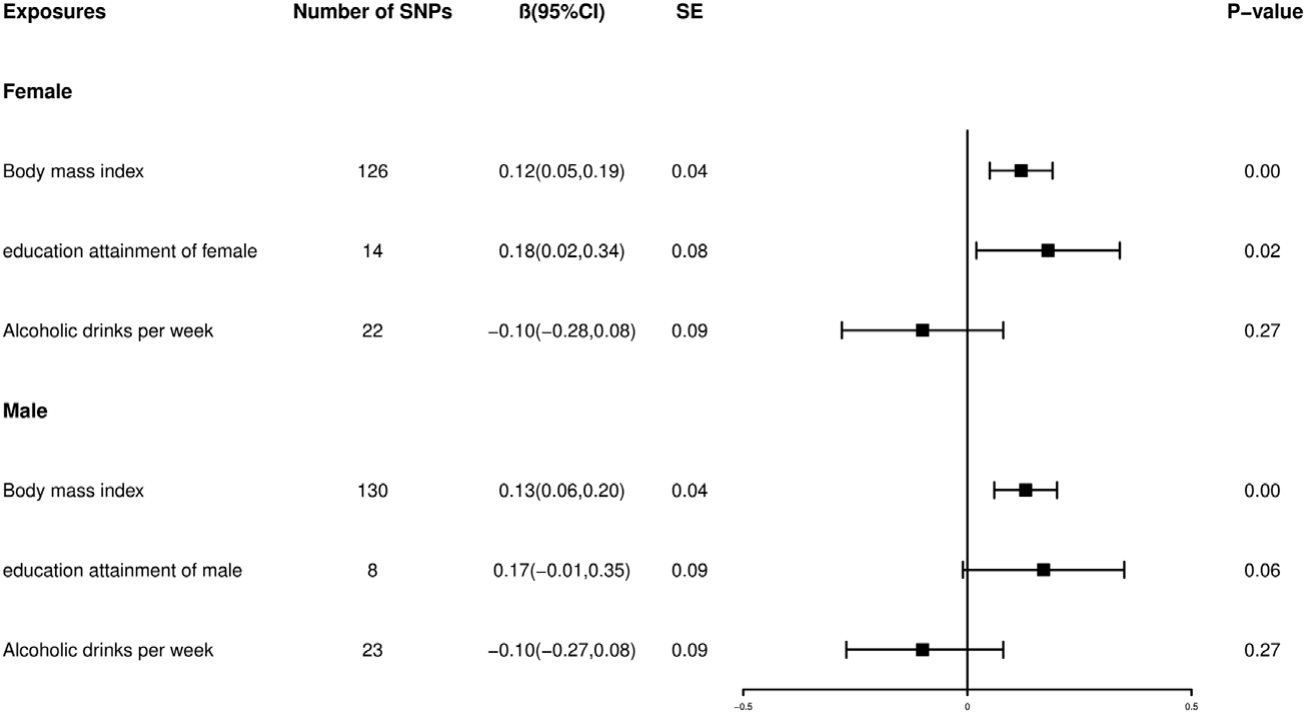
Forest plot showing the Multivariable mendelian randomization estimates adjusted for alcoholic drinks per week and BMI by IVW approach.

In the MVMR analysis (Figure 6), containing the SNPs associated with BMI (Female: *β* = 0.12, 95% CI: 0.05 to 0.19, *p* < 0.001; Male: *β* = 0.13, 95% CI: 0.06 to 0.20, *p* < 0.001, for the IVW method) and alcohol consumption (Female: *β* = −0.10, 95% CI: 0.28 to 0.08, *p* = 0.27; Male: *β* = −0.10, 95% CI: 0.27 to 0.08, *p* = 0.27, for the IVW method), the genetically predicted association between female EA and offspring BW maintained the same pattern (*β* = 0.18, 95% CI: 0.02 to 0.34, *p* = 0.02 for the IVW method) compared with univariable MR (*β* 0.24, 95% CI: 0.10 to 0.37, *p* < 0.001 for the IVW method). However, the association between genetically predicted male EA and offspring BW was attenuated (*β* = 0.17, 95% CI: 0.01 to 0.35, *p* = 0.06 for the IVW method) compared with univariable MR (*β* = 0.21, 95% CI: 0.07 to 0.34, *p* = 2.82 × 10–^3^ for the IVW method). The results regarding the association between male EA and offspring BW were inconsistent between univariable MR and MVMR, which could be explained by the fact that the observed changes in offspring BW caused by male EA may be (at least partially) confounded by paternal alcohol consumption and BMI.

### Two-step Mendelian randomization

**FIGURE 7:**
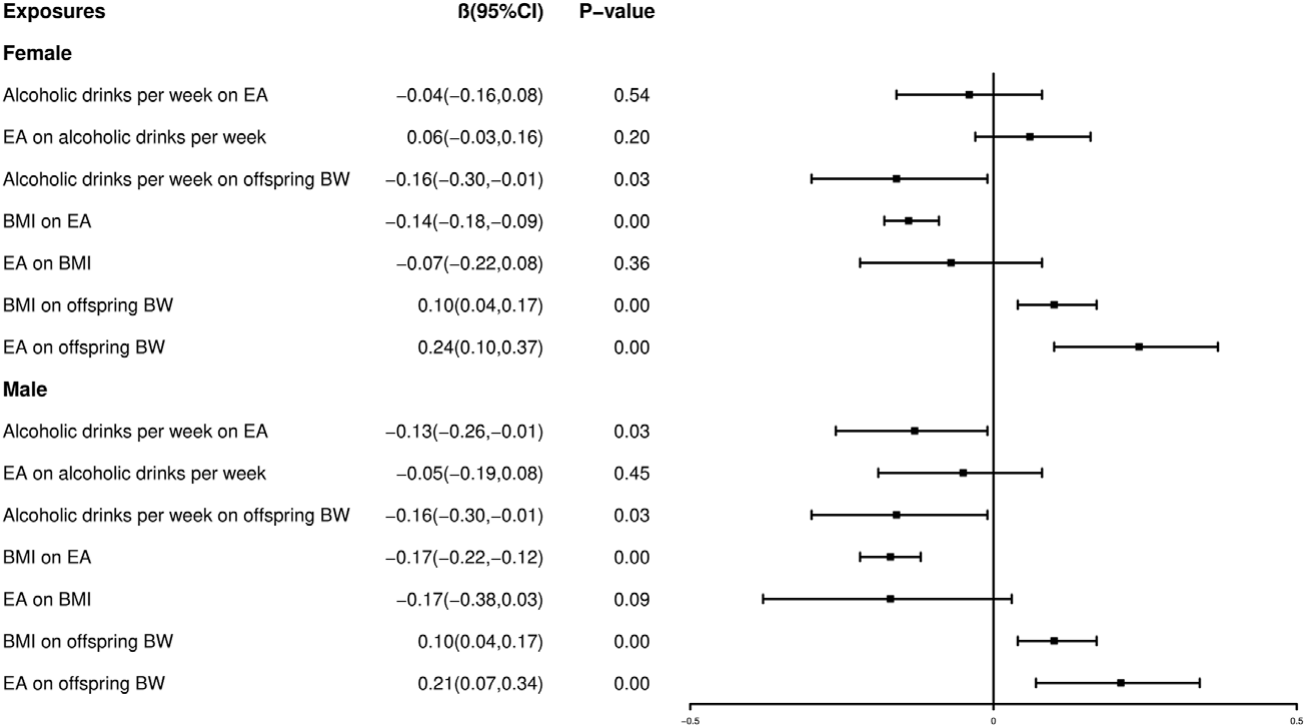
Forest plot showing the two-step MR with alcoholic drinks per week and BMI by IVW approach.

To further understand the role of alcoholic drinks per week and BMI in the causal association between EA and offspring birth weight, two-step MR was utilized in our study. In two-step MR (Figure 7), we found no evidence for a causal effect of EA on alcoholic drinks per week (Female: *β* = 0.06, 95% CI: -0.03 to 0.16, *p* = 0.20; Male: *β* = −0.05, 95% CI: −0.19 to 0.08, *p* = 0.45 for the IVW method) and BMI (Female: *β* = −0.07, 95% CI: −0.22 to 0.08, *p* = 0.36; Male: *β* = −0.17, 95% CI: −0.38 to 0.03, *p* = 0.09 for the IVW method), either in female or male. In reverse MR analysis, for female, we found no evidence of a causal association between alcoholic drinks per week and female EA (*β* = −0.04, 95% CI: −0.16 to 0.08, *p* = 0.54 for the IVW method); however, for male, alcoholic drinks per week was shown to be causally associated with both male EA (*β* = −0.13, 95% CI: −0.26 to −0.01, *p* = 0.03 for the IVW method) and offspring BW (*β* = −0.16, 95% CI: −0.30 to −0.01, *p* = 0.03 for the IVW method). Similarly, BMI was shown to be causally associated with both EA (Female: *β* = -0.14, 95% CI: 0.18 to -0.09, *p* < 0.001; Male: *β* = −0.17, 95% CI: −0.22 to −0.12, *p* < 0.001 for the IVW method) and offspring BW (*β* = 0.10, 95% CI: 0.04 to 0.17, *p* < 0.001 for the IVW method).

## Discussion

In this comprehensive MR study, we found evidence that the offspring BW was positively causally affected by female EA; specifically, an increase of 3.6 years of additional EA was associated with an increase in BW of approximately 116.16 g. The causal association between male EA and offspring BW was attenuated when we adjusted for alcoholic drinks per week and BMI using multivariable MR analysis, the two-step MR results then highlighted that the association between male EA and offspring BW may be confounded by traditional influencing factors of offspring BW (such as alcoholic drinks per week and BMI). We did not find a significant causal effect of EA on BW. The fetal origin of adult diseases (FOAD) hypothesis states that BW influences the long-term development of disease in adulthood (Wang et al., 2020). It is meaningful to explore potential exposures of birth weight. This result reminds us that we need to increase the level of personal education and actively carry out relevant public health activities, particularly among developing countries. For example, Brazil has one of the highest rates of low birth weight (approximately 8.0%) in developing countries over the past 10 years (United Nations Children’s Fund—UNICEF, https://data.unicef.org/topic/nutrition/low-birthweight/(2019).

Conclusions from previous epidemiological studies on the association between education and birth weight may be uncertain and sometimes conflicting. For instance, in the NICE cohort of 5,597 women, infants born to college-educated mothers were heavier than women with <12 years of education (Ogge et al., 2022). However, infants born to higher educated mothers (≥12 years of schooling) have significantly lower mean weights (Silvestrin et al., 2020). When motherly factors were included in some studies, the effect of fathers’ education on birth weight diminished (Mortensen, 2013; Leung et al., 2016). Our study further supports the positive causal association between female EA and BW. The results of previous studies showed that higher parents educated, and lower BMI (Vejrup et al., 2022). Couples with BMI ≥25 kg/m^2^ had higher odds of LGA than those with BMI <25 kg/m^2^ (Chen R. et al., 2021). In our study, we found a significant negative causal association between BMI and EA, and for male, BMI shows a more significant positive effect on offspring BW than male EA in the result of multivariable MR. These results suggest that, for males, intervening in these traditional influencing factors may reduce cases of low offspring BW attributable to lower levels of education.

The MR method has unique advantages because genes have been determined at birth, and SNPs as an IV will not be affected by various confounding factors. The reasonable temporal sequence guarantees the reliability of conclusions in causal inference. The causal estimation will be effective when the three assumptions in the MR model are satisfied. For this study, first, significantly correlated and independent SNP loci were selected that were closely related to female and male educational attainment. At the same time, the F-statistic of each SNP was greater than 10 (Supplementary Table S1; Supplementary Table S2), which indicates that the selected SNPs were robust IVs. Second, the data in this study were from the European population, which avoided the bias caused by different populations to a certain extent. Third, a series of sensitivity analyses further supported that there was no pleiotropy or heterogeneity in our results. Furthermore, we also conducted MVMR analysis to adjust for potential pleiotropy caused by other confounders. Therefore, the selected IVs and the study results were reliable.

Our study has some limitations. First, the participants in this study were all of European ancestry, but genetic inheritance patterns may vary across ancestry, so it may not be appropriate to generalize this finding to non-European ancestry. Given these limitations, the causal relationships suggested by our MR analysis should be interpreted with caution. More research is needed to better clarify the causal relationship between EA and offspring BW. Nevertheless, this study used two GWAS databases and lacked individual data to allow subgroup analysis by age or sex; thus, any potential nonlinear relationships or stratification effects by health status, age, or sex cannot be examined. Second, we only adjusted for BMI and alcohol consumption in the multivariable MR analysis, and some factors, such as age and gestational age, may confuse the association between education and birth weight (Mohammed et al., 2019; Jeena et al., 2020; Annan et al., 2021). If possible, studies should continue to explore other confounding factors to fully understand the relationship between education and birth weight.

## Conclusion

Generally, our study offered powerful evidence to suggest that higher EA in mothers plays a causal role in increasing offspring BW. In addition, the association between male EA and offspring BW may be confounded by traditional influencing factors of offspring BW (such as alcoholic drinks per week and BMI). Furthermore, more work should be implemented to clarify the potential mechanisms that may confound the association between education and BW.

## Data Availability

The original contributions presented in the study are included in the article/Supplementary Material, further inquiries can be directed to the corresponding author.

## Abbreviations

EA, Educational attainment; BW, Birth weight; MR, Mendelian randomization; SNP, single nucleotide polymorphism; GWAS, Genome-wide association studies; CI, Confidence intervals; IVW, Inverse variance weighted; MR-PRESSO, MR pleiotropy residual sum and outlier; WM, Weighted median estimate; OR, Odds ratio; BMI, Body mass index; MVMR, Multivariable Mendelian randomization.
