# Supplementary material for: Educational attainment and offspring birth weight: A bidirectional Mendelian randomization study
Source: Front Genet. 2022 Sep 1;13:922382. doi: 10.3389/fgene.2022.922382 (PMC9682907; doi:10.3389/fgene.2022.922382)
Supplement: Supplementary file 1 [file DataSheet3.docx]

**Supplementary Tables**

**Supplementary Table 1**: Effect estimates of the associations between genetic instruments of female’s education attainment and offspring’s birth weight.

| **SNP** | **Chr** | **Pos** | **EA** | **NEA** | **EAF** | ***F*** | **SNP-education** | | |  | **SNP-offspring BW** | | |
| --- | --- | --- | --- | --- | --- | --- | --- | --- | --- | --- | --- | --- | --- |
|  |  |  |  |  |  |  | ***β*** | **SE** | ***P*** |  | ***β*** | **SE** | ***P*** |
| rs1106761 | 8 | 142619234 | A | G | 0.36 | 39.51 | -0.023 | 0.003 | 2.73E-11 |  | -0.0123 | 0.0067 | 6.60E-02 |
| rs11140948 | 9 | 88001092 | A | C | 0.56 | 28.83 | -0.019 | 0.003 | 7.50E-09 |  | -0.0103 | 0.0064 | 1.10E-01 |
| rs11191270 | 10 | 104124605 | T | C | 0.20 | 32.59 | -0.025 | 0.004 | 1.96E-09 |  | -0.0176 | 0.0079 | 2.60E-02 |
| rs1267487 | 6 | 14720375 | A | G | 0.76 | 36.43 | -0.025 | 0.004 | 3.72E-08 |  | 0.0099 | 0.0084 | 2.40E-01 |
| rs1790123 | 12 | 123659542 | T | C | 0.77 | 41.54 | -0.027 | 0.004 | 8.06E-11 |  | -0.0142 | 0.0067 | 3.30E-02 |
| rs34305371 | 1 | 72733610 | A | G | 0.09 | 33.60 | 0.036 | 0.006 | 7.89E-10 |  | 0.0145 | 0.0106 | 1.70E-01 |
| rs3852788 | 16 | 72225786 | T | C | 0.40 | 28.15 | 0.019 | 0.003 | 3.50E-08 |  | 0.0081 | 0.0054 | 1.40E-01 |
| rs4445276 | 9 | 1755535 | A | G | 0.26 | 27.33 | 0.021 | 0.004 | 1.83E-08 |  | 0.0074 | 0.0068 | 2.80E-01 |
| rs4732129 | 7 | 135225156 | C | T | 0.32 | 25.65 | -0.019 | 0.004 | 2.96E-08 |  | -0.0042 | 0.0068 | 5.40E-01 |
| rs4744251 | 9 | 96272624 | T | C | 0.33 | 28.67 | 0.02 | 0.004 | 1.90E-08 |  | -0.0048 | 0.0067 | 4.80E-01 |
| rs61160187 | 5 | 60111579 | G | A | 0.38 | 27.58 | 0.019 | 0.003 | 4.86E-08 |  | -0.0026 | 0.0065 | 6.80E-01 |
| rs61817482 | 1 | 204570347 | A | G | 0.21 | 33.26 | 0.025 | 0.004 | 1.60E-09 |  | -0.0101 | 0.008 | 2.10E-01 |
| rs6456714 | 6 | 26335346 | G | C | 0.60 | 28.06 | 0.019 | 0.003 | 4.03E-08 |  | -0.0021 | 0.0066 | 7.50E-01 |
| rs6750720 | 2 | 100670745 | G | C | 0.37 | 36.46 | 0.022 | 0.004 | 1.55E-10 |  | 0.0094 | 0.0057 | 9.80E-02 |
| rs6839705 | 4 | 106144735 | C | A | 0.64 | 29.87 | -0.02 | 0.003 | 1.44E-08 |  | -0.0134 | 0.0056 | 1.60E-02 |
| rs6882046 | 5 | 87968864 | G | A | 0.31 | 40.17 | 0.024 | 0.004 | 4.67E-10 |  | 0.0098 | 0.0061 | 1.10E-01 |
| rs7613360 | 3 | 49916710 | T | C | 0.40 | 60.96 | -0.028 | 0.003 | 1.42E-16 |  | -0.0058 | 0.0065 | 3.80E-01 |
| rs7701853 | 5 | 120154333 | G | A | 0.32 | 25.58 | -0.019 | 0.003 | 2.62E-08 |  | 6.00E-04 | 0.0068 | 9.30E-01 |
| rs7868984 | 9 | 23357826 | C | T | 0.42 | 45.58 | 0.024 | 0.003 | 2.90E-12 |  | 0.0062 | 0.0065 | 3.40E-01 |
| rs795980 | 4 | 140801666 | G | A | 0.33 | 38.03 | 0.023 | 0.004 | 1.40E-10 |  | 0.0082 | 0.0058 | 1.60E-01 |
| rs9320913 | 6 | 98584733 | A | C | 0.50 | 59.08 | 0.027 | 0.003 | 1.11E-15 |  | -0.0027 | 0.0054 | 6.20E-01 |
| rs9527702 | 13 | 58384392 | G | A | 0.24 | 39.61 | -0.026 | 0.004 | 1.03E-12 |  | -0.004 | 0.0071 | 5.70E-01 |

SNP, single-nucleotide polymorphism id; Chr, chromosome; Pos, Position, genome position in base pair; EA, effect allele and alternative allele; EAF, effective allele frequency; β, SNP effect size; SE, standard error; P, F represent p-value, and F-statistic, respectively.

**Supplementary Table 2**: Effect estimates of the associations between genetic instruments of male’s education attainment and offspring’s birth weight.

| **SNP** | **Chr** | **Pos** | **EA** | **EAF** | ***F*** | **SNP-education** | | |  | **SNP-offspring BW** | | |
| --- | --- | --- | --- | --- | --- | --- | --- | --- | --- | --- | --- | --- |
|  |  |  |  |  |  | ***β*** | **SE** | **P** |  | ***β*** | **SE** | ***P*** |
| rs10740118***** | 10 | 65101207 | C | 0.42 | 31.27 | 0.022 | 0.004 | 4.51E-09 |  | 0.0039 | 0.0054 | 4.70E-01 |
| rs11123820 | 2 | 100871186 | G | 0.39 | 42.30 | 0.026 | 0.004 | 1.82E-11 |  | 0.0027 | 0.0066 | 6.80E-01 |
| rs11712056 | 3 | 49914397 | C | 0.44 | 51.12 | -0.028 | 0.004 | 1.58E-13 |  | -0.0069 | 0.0054 | 2.00E-01 |
| rs12410444 | 1 | 44188719 | G | 0.30 | 25.80 | 0.022 | 0.004 | 2.76E-08 |  | 0.0021 | 0.007 | 7.60E-01 |
| rs12436179 | 14 | 41368530 | A | 0.63 | 29.88 | -0.022 | 0.004 | 1.60E-08 |  | -0.0023 | 0.0066 | 7.30E-01 |
| rs12462428 | 19 | 16694610 | C | 0.21 | 26.18 | -0.026 | 0.005 | 1.93E-08 |  | -0.0024 | 0.0068 | 7.20E-01 |
| rs1572198 | 13 | 58330048 | T | 0.28 | 24.22 | -0.023 | 0.004 | 2.87E-08 |  | -0.0131 | 0.006 | 2.90E-02 |
| rs2553520 | 5 | 113860627 | C | 0.24 | 34.21 | -0.026 | 0.004 | 1.41E-09 |  | 0.006 | 0.0072 | 4.10E-01 |
| rs34305371 | 1 | 72733610 | A | 0.10 | 30.43 | 0.038 | 0.007 | 4.51E-09 |  | 0.0145 | 0.0106 | 1.70E-01 |
| rs35942385 | 2 | 144208523 | T | 0.37 | 26.65 | 0.021 | 0.004 | 3.16E-08 |  | 0.0061 | 0.0066 | 3.60E-01 |
| rs3847223 | 9 | 1790488 | T | 0.52 | 34.59 | 0.023 | 0.004 | 6.98E-10 |  | 0.0077 | 0.0053 | 1.50E-01 |
| rs62263923 | 3 | 85674790 | G | 0.35 | 26.65 | 0.021 | 0.004 | 4.42E-08 |  | 0.0049 | 0.0067 | 4.70E-01 |
| rs7029201 | 9 | 23358081 | A | 0.41 | 50.43 | 0.028 | 0.004 | 1.65E-13 |  | 0.0062 | 0.0065 | 3.40E-01 |
| rs9372625 | 6 | 98344031 | A | 0.39 | 43.10 | 0.026 | 0.004 | 2.37E-11 |  | 6.00E-04 | 0.0055 | 9.20E-01 |
| rs9739070 | 12 | 123771032 | G | 0.80 | 29.12 | -0.025 | 0.004 | 1.68E-08 |  | -0.0127 | 0.008 | 1.10E-01 |

SNP, single-nucleotide polymorphism id; Chr, chromosome; Pos, Position, genome position in base pair; EA, effect allele and alternative allele; EAF, effective allele frequency; β, SNP effect size; SE, standard error; P, F represent p-value, and F-statistic, respectively.

*The SNP is with a palindrome structure, and was therefore excluded from the IVs

**Supplementary Table 3:** Evidence of association (*P* < 5×10^-8^) of the 4 SNPs used as genetic instruments from the GWAS for Mendelian randomization analyses of female's EA and offspring BW with secondary phenotypes.

| **SNP** | | | **Chr** | | | **Gene** | | | **Diseases & traits** | |
| --- | --- | --- | --- | --- | --- | --- | --- | --- | --- | --- |
| rs1106761 | | | 8 | | | AC138647.1 | | | Alcohol intake frequency (UK biobank), Intelligence multi trait analysis (PMID:29326435), Weight (UK biobank), Body mass index (UK biobank), Waist circumference (UK biobank), Body fat percentage (UK biobank), Leg fat percentage left (UK biobank) | |
| rs7613360 | | | 3 | | | ACTBP13 | | | Body mass index (UK biobank)， Weight (UK biobank)， Time spent watching television (UK biobank)， High light scatter reticulocyte count (UK biobank)， Whole body fat-free mass (UK biobank)， Job involves heavy manual or physical work (UK biobank)， Trunk fat-free (UK biobank)， Reticulocyte fraction of red cells (UK biobank)， Job involves mainly walking or standing (UK biobank)， Job involves heavy manual or physical work (UK biobank) | |
| rs6882046 | | | 5 | | | LINC00461 | | | Intelligence multi-trait analysis (PMID:29326435), Neuroticism (PMID:29255261), Body mass index (PMID: 28892062), Alcohol intake frequency (UK biobank), Average weekly red wine intake (UK biobank), Trunk fat percentage (UK biobank), Job involves mainly walking or standing (UK biobank), Irritability (UK biobank), Job involves heavy manual or physical work (UK biobank), Body fat percentage (UK biobank) | |
| rs1790123 | | | 12 | | | MPHOSPH9 | | | Time spent watching television (UK biobank), No blood clot (UK biobank), bronchitis (UK biobank), Hip circumference adjusted for BMI (PMID:25673412), Height (PMID:20881960), Plateletcrit (PMID:27863252), Diastolic blood pressure (UK biobank), Trunk fat-free mass (UK biobank) | |
|  |  | | |  | | |  | | | |
| Abbreviations: Chr, chromosome; SNP, single nucleotide polymorphism. | | | | | | | | | | |
| a Traits associated with the SNP according to previous genome-wide association studies. | | | | | | | | | | |
| **Supplementary Table 4:** Evidence of association (*P*<5*10^-8^) of the 3 SNPs used as genetic instruments from the GWAS for Mendelian randomization analyses of male's EA and offspring BW with secondary phenotypes. | | | | | | | | | |  |
| **SNP** | | **Chr** | | | **Gene** | | | **Diseases & traits** | |  |
| rs11123820 | | 2 | | | LINC01104 | | | Leg fat mass left (UK biobank), Leg fat mass right (UK biobank), Waist circumference Diseases (UK biobank), Body mass index, Fluid intelligence score (UK biobank) | |  |
| rs11712056 | | 3 | | | ACTBP13 | | | Time spent watching television (UK biobank), Body mass index (UK biobank), Riculocyte fraction of red cells (PMID:27863252), Immature fraction of reticulocytes (PMID:27863252), Inflammatory bowel disease (PMID:26192919), Age at last live birth (UK biobank), Age first birth (PMID:27798627) | |  |
| rs62263923 | | 3 | | | CADM2 | | | Body mass index (UK biobank), Ever smoked (UK biobank), Past tobacco smoking (UK biobank) | |  |

| Abbreviations: Chr, chromosome; SNP, single nucleotide polymorphism.  a Traits associated with the SNP according to previous genome-wide association studies. |
| --- |

**Supplementary Figures**


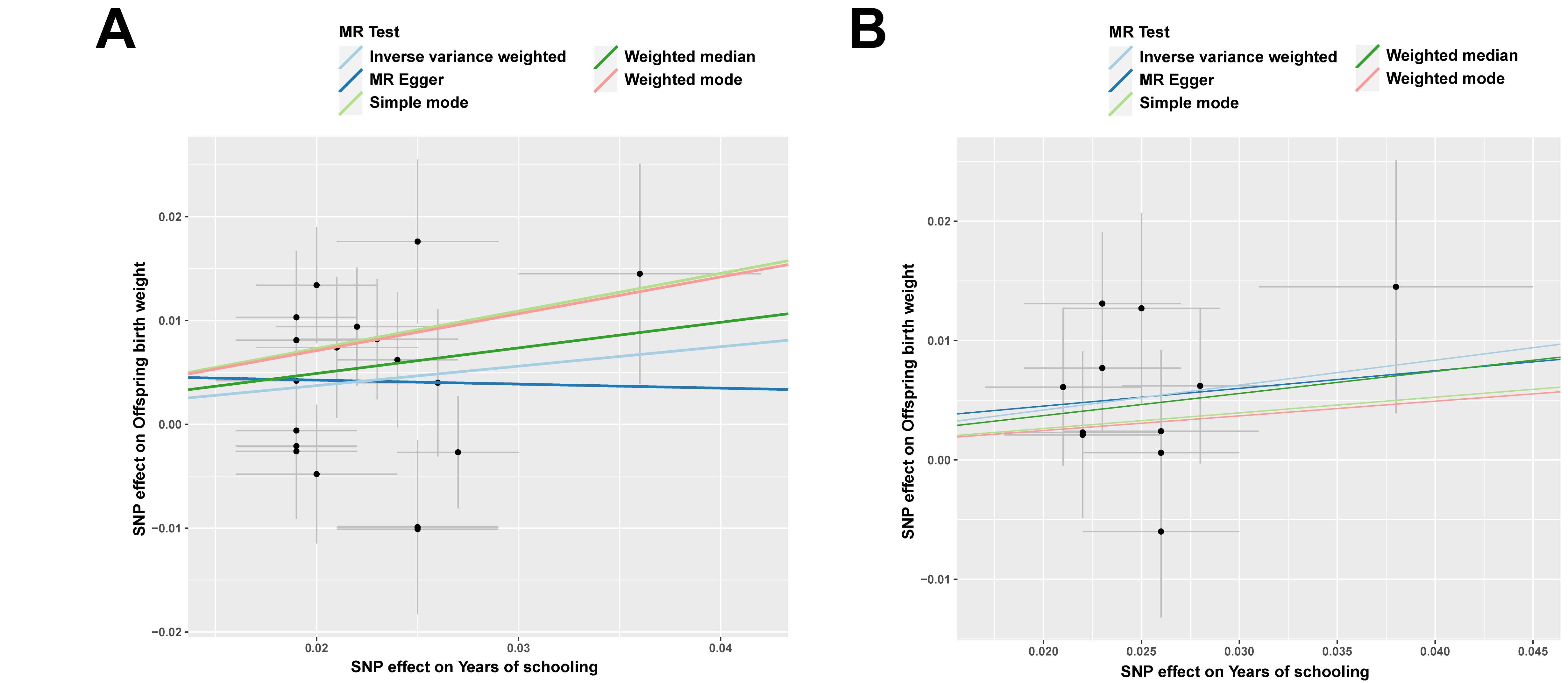


**Supplementary Figure 1.** **The results of the Mendelian randomization analysis after excluding SNPs associated with secondary phenotypes at genome-wide significance levels.** The causal estimate for the effect of female’s educational attainment on offspring birth weight after excluding SNPs associated with secondary phenotypes at genome-wide significance levels was shown in (**A**), while the overall effect for the casual association male’s educational attainment with offspring’s birth weight after excluding SNPs associated with secondary phenotypes at genome-wide significance levels was presented in (**B**).





**Supplementary Figure 2.** **Forest plot showing mendelian randomization estimates of the associations between education attainment and offspring birth weight after excluding SNPs associated with secondary phenotypes at genome-wide significance levels by different methods.** CI, Confidence interval; MR, Mendelian randomization.
